# Understanding Citizens’ Response to Social Activities on Twitter in US Metropolises During the COVID-19 Recovery Phase Using a Fine-Tuned Large Language Model: Application of AI

**DOI:** 10.2196/63824

**Published:** 2025-02-11

**Authors:** Ryuichi Saito, Sho Tsugawa

**Affiliations:** 1 Institute of Systems and Information Engineering University of Tsukuba Tsukuba Japan

**Keywords:** COVID-19, restriction, United States, X, Twitter, sentiment analysis, large language model, LLM, GPT-3.5, fine-tuning

## Abstract

**Background:**

The COVID-19 pandemic continues to hold an important place in the collective memory as of 2024. As of March 2024, >676 million cases, 6 million deaths, and 13 billion vaccine doses have been reported. It is crucial to evaluate sociopsychological impacts as well as public health indicators such as these to understand the effects of the COVID-19 pandemic.

**Objective:**

This study aimed to explore the sentiments of residents of major US cities toward restrictions on social activities in 2022 during the transitional phase of the COVID-19 pandemic, from the peak of the pandemic to its gradual decline. By illuminating people’s susceptibility to COVID-19, we provide insights into the general sentiment trends during the recovery phase of the pandemic.

**Methods:**

To analyze these trends, we collected posts (N=119,437) on the social media platform Twitter (now X) created by people living in New York City, Los Angeles, and Chicago from December 2021 to December 2022, which were impacted by the COVID-19 pandemic in similar ways. A total of 47,111 unique users authored these posts. In addition, for privacy considerations, any identifiable information, such as author IDs and usernames, was excluded, retaining only the text for analysis. Then, we developed a sentiment estimation model by fine-tuning a large language model on the collected data and used it to analyze how citizens’ sentiments evolved throughout the pandemic.

**Results:**

In the evaluation of models, GPT-3.5 Turbo with fine-tuning outperformed GPT-3.5 Turbo without fine-tuning and Robustly Optimized Bidirectional Encoder Representations from Transformers Pretraining Approach (RoBERTa)–large with fine-tuning, demonstrating significant accuracy (0.80), recall (0.79), precision (0.79), and *F*_1_-score (0.79). The findings using GPT-3.5 Turbo with fine-tuning reveal a significant relationship between sentiment levels and actual cases in all 3 cities. Specifically, the correlation coefficient for New York City is 0.89 (95% CI 0.81-0.93), for Los Angeles is 0.39 (95% CI 0.14-0.60), and for Chicago is 0.65 (95% CI 0.47-0.78). Furthermore, feature words analysis showed that COVID-19–related keywords were replaced with non–COVID-19-related keywords in New York City and Los Angeles from January 2022 onward and Chicago from March 2022 onward.

**Conclusions:**

The results show a gradual decline in sentiment and interest in restrictions across all 3 cities as the pandemic approached its conclusion. These results are also ensured by a sentiment estimation model fine-tuned on actual Twitter posts. This study represents the first attempt from a macro perspective to depict sentiment using a classification model created with actual data from the period when COVID-19 was prevalent. This approach can be applied to the spread of other infectious diseases by adjusting search keywords for observational data.

## Introduction

### Background

The global SARS-CoV-2 outbreak, which began in 2020, remains vivid in the collective memory of humanity as of 2024. It is beyond dispute that it was an unprecedented pandemic in human history. For >3 years since the World Health Organization declared the novel COVID-19 a Public Health Emergency of International Concern on January 30, 2020 [1,2], humanity has grappled with COVID-19 as a major social challenge. As of March 2024, the total number of COVID-19 cases has surpassed 676 million, >6 million people have died, and >13 billion vaccine doses have been administered [3]. To assess the scale and severity of the COVID-19 pandemic, it is crucial to confirm public health indicators such as these. While numerous studies have been undertaken to address the pandemic, from artificial intelligence and medical imaging to printing technology [4], it is also important to conduct sociopsychological observations to understand how citizens have perceived the COVID-19 pandemic.

We assess public sentiment during the pandemic by analyzing social media posts using natural language processing (NLP) techniques, which have significantly advanced since the 2010s. An NLP-based approach can address the limitations of traditional social science research methods, which are often constrained by limited observational data and the potential for indirect bias from nonrespondents [5]. Previous studies that analyzed sentiment during the COVID-19 pandemic can be categorized into 2 main groups. One group focused its investigations on the initial outbreak that occurred in March 2020 [6-10], while the other focused on specific thematic areas, such as vaccination efforts [11-14]. However, no study to date has captured the cyclic fluctuations in social sentiment from macro and long-term perspectives because these studies are based on data collected using keywords related to COVID-19, and these words usually reflect negative feelings. Furthermore, despite variations in the spread of COVID-19 between urban and rural areas [15], many studies have focused on sentiment analysis at the level of language or country, thereby limiting the extraction of insights considering local infection status, which is crucial from a public health perspective.

Our previous study [16] analyzed social media posts about citizens’ activities that were constrained by the pandemic, and the degree of sentiment changed depending on the context of the source observational data instead of COVID-19–related texts. In addition, we focused on major American cities such as New York City, Los Angeles, and Chicago, which have similar social conditions. Using this approach, we addressed the problems of previous studies and revealed the general trends of citizens’ sentiments from December 2019 to December 2021 during the COVID-19 pandemic.

This study further develops this research and examines the period from December 2021, when the Omicron variants surged, to December 2022, a period when public interest in COVID-19 generally waned. In the United States in 2021, outbreaks caused by the Omicron variant strain continued intermittently, starting with BA.1 at the end of 2021, followed by BA.2 and BA.3 [17], prompting state governments to issue repeated warnings and subsequently rescind them. The World Health Organization’s Public Health Emergency of International Concern ended in May 2023 [2], and thus, 2022 can be considered a transition period from the pandemic period to the postpandemic period. We believe it is important to observe citizens’ sentiments in the United States during this period. In addition, because previous research involving sentiment classification models relied on lexicon dictionaries or training datasets before 2020 when SARS-CoV-2 emerged, the domain adaption between the lexicon dictionary or training data (source domain) and the observational data (target domain) [18] was not sufficiently considered in NLP. To address this problem, we constructed a sentiment classification model fine-tuned using data extracted under conditions identical to those of the actual observational dataset. Using these methodologies, we propose a definitive approach for estimating social sentiment during the COVID-19 pandemic.

### Objectives

Sentiment classification methods based on algorithms can be broadly divided as follows: lexicon-based approaches that infer text sentiment from tokens that have been assigned sentiment scores in advance and machine learning approaches that infer text sentiment from models trained on datasets [19]. There are several studies on lexicon-based sentiment analysis during a crisis. A study in Wuhan, China, tracked the change in public sentiment during the first 12 weeks after the identification of COVID-19 by examining posts on Weibo. The study revealed the pattern of trajectories—from confusion and fear, through disappointment, frustration, depression, and anxiety, and finally to happiness and gratitude—using the Emotion Vocabulary of the Dalian University of Technology [7]. A study in Europe and the United States measured the sentiment toward immigration by examining related tweets in Germany, Italy, Spain, the United Kingdom, and the United States during the early stages of the COVID-19 pandemic. The study estimated the sentiment scores of tweets, using Valence Aware Dictionary and Sentiment Reasoner, and revealed various themes regarding immigration for each country through topic modeling [20].

Some studies on sentiment analysis during crises use machine learning approaches. An analysis in the United States investigated the communication patterns of provaccine and antivaccine users on Twitter (now called X) by visualizing a retweet network related to the measles, mumps, and rubella vaccine during the 2015 California Disneyland measles outbreak. The study classified the users into provaccination, antivaccination, and neutral groups using a support vector machine. The results showed that most of the users were overwhelmingly provaccination, while antivaccine users resided in their own enclosed structural community [21]. A study in the United States also analyzed public perceptions of gig work on Twitter for 2 weeks before and after the COVID-19 emergency declaration. The study trained a machine learning model based on 10 different labels. The results showed that tweets reflected an increased sense of community and concern toward gig workers during the pandemic [22]. An investigation on Instagram demonstrated the changes in hate and misinformation against East Asians that occurred through Meta’s content moderation early in the COVID-19 pandemic. The study used supervised machine learning methods to estimate text emotions associated with human faces in posts with the hashtag #coronavirus [8]. A study on Twitter and Weibo revealed a pattern in which negative sentiment peaked before the 2020 lockdowns in many countries, followed by a gradual recovery in sentiment [9]. A study in China examined the psychological impacts of the COVID-19 epidemic declaration on individuals using Weibo data. Findings revealed an increase in negative emotions, such as anxiety and depression, alongside a decrease in life satisfaction and positive emotions using the pretrained psychological prediction model [10].

The disadvantage of lexicon-based approaches is that they are highly domain oriented [19]. Then, to predict sentiment in a specific domain, such as the discourse space of Twitter during the COVID-19 pandemic, a machine learning–based approach using training data, especially a neural network approach, is reasonable in terms of accuracy. This approach involves Transformer-based models with attention mechanisms [23], which assign weights to tokens based on their relationships, without processing time-series data sequentially, as is done in natural language. Language models such as GPT-1 [24] and Bidirectional Encoder Representations from Transformers (BERT) [25], which leverage the Transformer architecture and have undergone extensive pretraining on large datasets, have become readily available tools for NLP tasks.

A study on Twitter examined how users with different ideological views and follower bases expressed vaccine favorability and specific vaccine-related concerns. Users’ perception of the vaccine was classified by a fine-tuned BERT model using training data that were coded by the authors. The results from linear mixed-effects models showed a contrast in vaccines between conservative and liberal users, and users with large numbers of followers tended to be more favorable toward vaccines, while those with an average number of followers were prone to be more concerned about vaccines [11]. A study from January 2021 to January 2022 on Twitter used a change-point detection algorithm to identify significant shifts in public sentiment regarding the pandemic. Validation of these findings was accomplished by cross-referencing with contemporaneous news reports. Furthermore, the estimation used BERT fine-tuned with labeled tweets from Kaggle to gauge public attitudes [26]. A study on Twitter and Reddit investigates public sentiment regarding COVID-19 vaccines from January 2020 to March 2022. Using a fine-tuned DistilRoBERTa model, augmented with back-translation, it reveals that Twitter sentiment was predominantly negative, whereas Reddit sentiment was mostly positive [13]. A study in the United Kingdom used a hybrid model, combining Valence Aware Dictionary and Sentiment Reasoner, TextBlob, and BERT, to analyze public perceptions of COVID-19 contact tracing apps on Twitter and Facebook. Results indicated varying sentiments influenced by debates on centralized versus decentralized data handling in app-based contact tracing [27].

In a language model based on the Transformer architecture, there is a pretraining stage in which initial parameters are set and a fine-tuning stage in which parameters are adapted for a specific target task or domain. Given that language models may not possess adequate pretraining on social discourse after major transformative events such as the COVID-19 pandemic, it is appropriate to fine-tune the model using training data with conditions similar to the observed data. Therefore, the following research question (RQ) initially guided our study: *To what extent can the performance of classification tasks be enhanced through the use of posts on Twitter during the pandemic as training data? (RQ1)*

In addition, since 2020, there have been advancements in large language models (LLMs) such as GPT-3, making them capable of not only understanding but also generating sentences [28]. In this paper, we use GPT-3.5 Turbo [29], which is the latest version that allows fine-tuning, and we ask the following question: *To what extent does GPT-3.5 Turbo enhance the performance of sentiment classification tasks in comparison with conventional Transformer-based models? (RQ2)*

Previous studies have focused mainly on the several months of lockdown starting in April 2020 and the rollout of vaccinations from late 2020 onward, but no study has captured the long-term trend in social sentiment for specific regions during the COVID-19 pandemic period from 2020 to 2023. These tendencies simultaneously indicate the difficulty of analyzing public sentiment during the COVID-19 pandemic from a macro and diachronic perspective. We intend to undertake a long-term and macroscopic observation by targeting text that discusses the social activities of citizens restricted by lockdowns and similar measures implemented in response to the spread of COVID-19, rather than focusing solely on text explicitly containing keywords such as “COVID-19,” “virus,” and “lockdown,” as was the case in the previous studies. According to a study about adaptation to the “new normal,” the term “the new normal” first emerged during the 2008 financial crisis and was used again to point out how the COVID-19 pandemic had changed essential aspects of human life [30]. A study compared risk perception in a sample in April 2021 and January 2022, based on the assumption that the “new normal” involved ongoing restrictions starting with widespread vaccine access and the vaccination of populations considered vulnerable. The study found that people tend to overestimate COVID-19 risks, particularly for children and healthy individuals [31]. This study considers this “new normal” period in the time frame from December 2021 to December 2022 and asks the following question: *To what extent will the cyclical sentiment of citizens toward restricted activities in major US cities during the “new normal” period weaken over time following its peak in December 2021? (RQ3)*

Previous studies focused on observations from countries and language areas such as the English-speaking world. However, a geospatial study showed that the spread of COVID-19 in the United States differed between urban and rural areas even within the same state and that infection status was correlated with each county’s social vulnerability and community resilience [15]. Moreover, a study that surveyed rural and urban areas during the pandemic showed that rural residents were less sensitive to preventive health behaviors compared with urban residents due to the influence of political ideology or demographic factors [32]. On the basis of these findings, it is imperative to distinguish and analyze urban and rural areas separately, particularly within the US context. Therefore, in this study, we intentionally focused on major urban centers within the United States to estimate social sentiment and asked the following question: *In comparing citizens’ sentiments across major metropolitan areas in the United States, namely, New York City, Los Angeles, and Chicago, between December 2021 and December 2022, what differences or similarities in sentiment were there? (RQ4)*

## Methods

### Overview

We collected posts on Twitter related to restricted activities during the COVID-19 pandemic in New York City, Los Angeles, and Chicago from October 2021 to December 2022. Posts from October 2021 to November 2021 were used to train the sentiment classification model, while posts from December 2021 to December 2022 were used to estimate sentiment and extract feature words. Then, the correlation coefficient between estimated sentiment and actual infected cases was determined. In addition, the complete code for this study is available on the GitHub repository [33].

### Data Collection

In this study, we chose the 3 largest cities in the United States according to the US Census Bureau [34]: New York City, Los Angeles, and Chicago. Specifically, New York County, Los Angeles County, and Cook County were selected as observation targets. In the data collection process, the Full-archive Search Application Programming Interface (API) of Twitter API v2 [35] was used to retrieve messages posted within a 25-mile radius of the latitude and longitude of the city hall for each city. This radius is the upper limit of the Full-archive Search API, ensuring comprehensive coverage of the urban core. Our observation period was from December 2021, when the emergence of the Omicron variant led to record-high number of cases in the United States, to December 2022, when interest in COVID-19 had waned; this builds on the author’s previous study [16] that estimated sentiment in the same 3 cities from December 2019 to January 2022. Posts from October 2021 to November 2021 were also used as training data for neural network models.

The search string used for retrieving posts on Twitter uses keywords associated with citizens’ activities that were constrained by lockdowns and similar measures implemented in response to the COVID-19 pandemic, as described in the study by Saito and Haruyama [16]. These keywords are shown in Multimedia Appendix 1. The use of these specific keywords enables a coherent overview of the citizens’ psychological landscape from a macro perspective. These keywords are categorized by restriction type (ie, stay-at-home order, restrictions on gatherings, and travel restrictions) and are specified as arguments for the Twitter API version 2’s Search API, using “OR” conditions. In addition, retweets were excluded from the search, and only English posts were retrieved. The retrieved posts were processed to remove noise, including URLs, mentions, and hashtags, followed by text normalization and deduplication, before being used for training and classification with neural networks.

### Observational Data

We obtained posts from December 2021 to December 2022 using the Search API of Twitter API v2. The total number of posts was 119,437, and the total file size was 34.4 MB. Table 1 shows the number of posts by restriction type, and the number of unique users. Moreover, this data collection was conducted during the period from June 15, 2023, to June 22, 2023. For privacy protection, identifying information such as author IDs and usernames was removed, retaining only the text for analysis.

**Table 1:** Number of posts and unique users collected from Twitter between December 2021 and December 2022.

		New York City	Los Angeles	Chicago
**Posts, n (%)**				
	Stay-at-home order	12,969 (23.97)	9622 (21.98)	5308 (24.64)
	Restrictions on gatherings	14,095 (26.05)	13,448 (30.71)	5803 (26.94)
	Travel restrictions	27,045 (49.98)	20,715 (47.31)	10,432 (48.42)
	Total	54,109 (100.00)	43,785 (100.00)	21,543 (100.00)
Unique users, n		20,575^a^	17,588	8948

^a^For 113 tweets in New York City, unique users could not be identified because the author ID could not be obtained. Therefore, they were excluded from this table.

### Neural Network Model

To create a sentiment classifier, we used a Transformer-based neural network model using training data that are searched on the same data source, keywords, geolocation information, and language except for a period as actual observed data.

#### Training Data

As training data, we used Twitter posts obtained using Search API of Twitter API v2.0 under the same search keywords, geolocation, and language as observed data during the period from October 31, 2021, to November 27, 2021. This period was chosen to prevent any overlap with the observation data. The total number of results obtained was 3149, and the retrieved posts were preprocessed, such as noise removal, text normalization, and deduplication. For these pieces of data, an author and 2 Amazon Mechanical Turk (MTurk) workers labeled them as positive, negative, or neutral based on the instructions shown in Textbox 1, and the final label was determined by a majority vote. Tweets that could not be decided by a majority vote because each of the 3 people indicated different opinions were labeled as neutral. Concerning the instructions in Textbox 1, positive and negative sentiments were defined based on the available sentiment by [36], and neutral sentiments were defined by the authors according to the characteristics of the Twitter posts obtained. We also selected workers with master’s qualifications who have consistently been recognized for their high performance on Amazon MTurk. In addition, only texts were extracted for individual privacy measures, and due to Amazon MTurk’s inability to display 4-byte Unicode Transformation Format-8 emojis, we converted them to HTML spans in the task instructions, ensuring that the workers considered emojis when evaluating emotions. After labeling 3149 posts, the unanimous agreement rate among all 3 evaluators was 55.22% (1739/3149), and the 2-out-of-3 agreement rate was 28.74% (905/3149). The remaining 16.04% (505/3149) of posts were marked as neutral because they were of different values.

Labeled posts were shuffled and then filtered to extract 2400 instances for training the neural network model. These were then partitioned into training (1800/2400, 75%) and validation (600/2400, 25%) datasets. The remaining 749 instances were reserved for testing purposes. Table 2 shows the number of divided posts for each sentiment.

**Instructions for 3 evaluators to create training data and the prompt for GPT-3.5 Turbo for classifying sentiment. In the prompt in GPT-3.5 Turbo, this instruction was modified only to return numerical values such as positive to 0, neutral to 1, and negative to 2.**

**You are requested to perform sentiment classification (positive, negative, or neutral) for each tweet based on the following rules. These tweets have been collected for the purpose of scientific research.**

- Positive sentiments: admire, amazing, assure, celebration, charm, eager, enthusiastic, excellent, fancy, fantastic, frolic, graceful, happy, joy, luck, majesty, mercy, nice, patience, perfect, proud, rejoice, relief, respect, satisfactorily, sensational, super, terrific, thank, vivid, wise, wonderful, zest, expectations, etc.
- Negative sentiments: abominable, anger, anxious, bad, catastrophe, cheap, complaint, condescending, deceit, defective, disappointment, embarrass, fake, fear, filthy, fool, guilt, hate, idiot, inflict, lazy, miserable, mourn, nervous, objection, pest, plot, reject, scream, silly, terrible, unfriendly, vile, wicked, etc.
- Neutral sentiments: neither positive nor negative, such as text without sentiment, stating a fact, question, news article, advertisement, solicitation, request, quote, unintelligible text, etc.
- When the sentiment is mixed, such as expressing both joy and sadness, use your judgment and choose the more strongly expressed emotion.

**Table 2:** Training, validation, and test data for creating the neural network models.

Data types	Positive, n (%)	Neutral, n (%)	Negative, n (%)
Training data (n=1800)	648 (36)	515 (28.61)	637 (35.39)
Validation data (n=600)	225 (37.5)	159 (26.5)	216 (36)
Test data (n=749)	270 (36.05)	214 (28.57)	265 (35.38)

#### Training of the Neural Network Model

**Figure 1:**
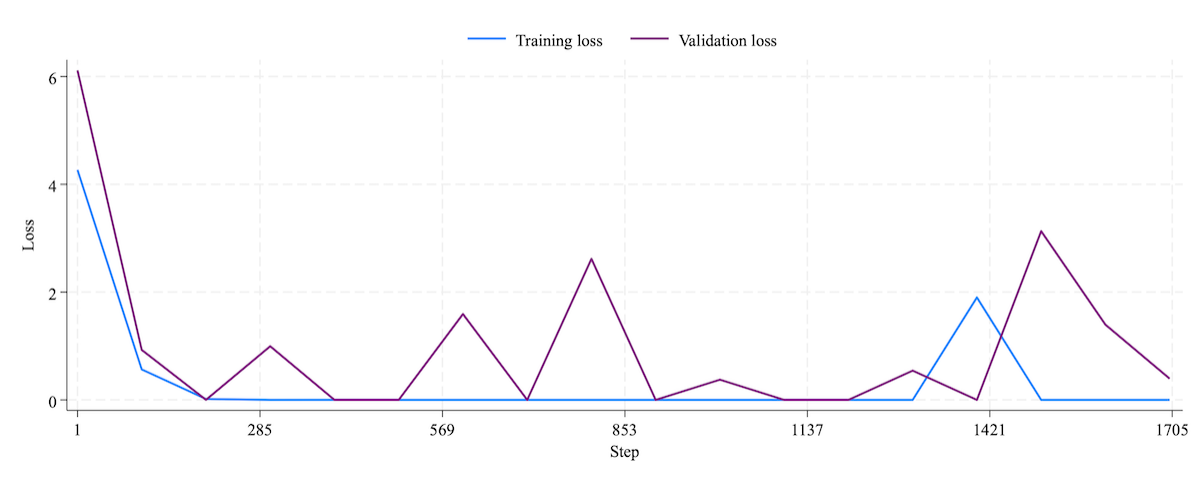
The learning curve of fine-tuning GPT-3.5 Turbo.

For sentiment classification in this research, we adopted GPT-3.5 Turbo, the latest version of GPT-3 that is trained using huge parameters and can be fine-tuned using real data. GPT-3.5 was trained on data up to September 2021, marking a distinction from GPT-3, which used training data through 2019. This difference suggests a substantial potential contribution to the contextual analysis of the globally prevalent COVID-19 pandemic since 2020. In the development of our model, we used gpt-3.5-turbo-1106 as the base model, subsequently fine-tuning it with the training data described in the Training Data section. The model was created using the following hyperparameters: a learning rate multiplier of 2, a batch size of 3, and a total of 3 epochs. These values were automatically optimized by the fine-tuning API based on the size of the training dataset. Figure 1 presents the learning curve, where the x-axis represents the number of steps and the y-axis represents the loss, which indicates how closely the model’s predictions align with the actual outcomes. The validation loss, which reflects the model’s performance on unseen data, decreases until approximately step 500 but then fluctuates intermittently up to step 1500, suggesting potential overfitting to the training data. Toward the final step, however, the validation loss decreases to 0.39, indicating that the model stabilizes and improves its generalization performance.

### Ethical Considerations

The study was conducted using publicly posted large volumes of Twitter data collected via the Twitter API, which is permitted only for academic research but does not involve any human subject research. Furthermore, these posts are not produced within a virtual community on Twitter. Data collection, creation of training and test datasets, and analysis were carried out following Twitter’s terms and conditions. In addition, all identifying information, such as author IDs, tweet IDs, display names, and usernames, was excluded, with only the tweet content used for the research. This study also ensures adherence to the guidelines outlined in the study by Luo et al [37].

## Results

### Evaluation of Sentiment Estimation Models

To evaluate the developed classification model, we compared it with the model Robustly Optimized BERT Pretraining Approach (RoBERTa)–large [38], which was fine-tuned using the same training data as the developed model, as well as GPT-3.5 Turbo, which was deliberately not fine-tuned. In addition, the following hyperparameters were used in fine-tuning a RoBERTa-large model: a learning rate of 5e-5, a batch size of 16 for training and 64 for validation, and a total of 3 epochs. In both GPT-3.5 Turbo and GPT-3.5 Turbo with fine-tuning, the same prompt (the role’s system parameter of the API) was set based on Textbox 1, with the only change being the modification to return numerical values, such as 0 for positive, 1 for neutral, and 2 for negative. Both models also implemented exception handling to skip the output as a value error if the generated text is not numeric. (There was only 1 value error in this evaluation when using GPT-3.5 Turbo with fine-tuning.)

Table 3 shows the performance evaluation of each model. Accuracy is the proportion of correctly predicted instances out of all the cases in the test data. Recall is the proportion of correctly predicted instances out of all actual instances for each class (positive, neutral, and negative). Precision is the proportion of correctly predicted instances out of all the cases predicted as each class. Although the neutral class recall was low for all models, with precision remaining relatively stable across all models, GPT-3.5 Turbo with fine-tuning still significantly outperformed the other models in the recall. This difference might have a significant impact on the accuracy difference. *F*_1_-score is a metric generally used to evaluate the performance of neural network models, and it provides a balanced assessment by considering both recall and precision. In both models, the *F*_1_-score value for the neutral label was much lower than the values for the other labels, which means that the subtle nuances of the boundaries between negative and neutral and between neutral and positive were not sufficiently trained. However, in both models, the *F*_1_-scores for the positive and negative labels exceeded 0.80, so we assume that there was no fatal effect on the results. Comparing the 2 models, RoBERTa-large and GPT-3.5 Turbo showed small differences in accuracy and average *F*_1_-score, but it can be said that GPT-3.5 Turbo without fine-tuning showed the same performance as RoBERTa-large with fine-tuning.

RQ1 asks to what extent the performance of classification tasks can be enhanced through the use of Twitter’s posts during the pandemic as training data. As shown in Table 3, GPT-3.5 Turbo with fine-tuning had a 5% higher accuracy and a 6% higher *F*_1_-score compared with GPT-3.5 Turbo without fine-tuning, which clearly demonstrates the improvement in performance because of fine-tuning. RQ2 asks to what extent GPT-3.5 Turbo enhances the performance of sentiment classification tasks compared with conventional Transformer-based models. GPT-3.5 Turbo with fine-tuning had a 4% higher accuracy and a 7% higher *F*_1_-score compared with RoBERTa-large with fine-tuning, which distinctly demonstrates the performance improvement because of large-scale learning. On the basis of the abovementioned results, this study used GPT-3.5 Turbo with fine-tuning.

Table 4 shows examples of sentiments classified and indexed by GPT-3.5 Turbo with fine-tuning. Each tweet is classified as –1 for positive, 0 for neutral, or 1 for negative. The higher the value of the index, the more pessimistic the sentiment is throughout the week, and the lower the value, the more optimistic the sentiment.

**Table 3:** Performance evaluation of the neural network models for sentiment classification.

Model and class		Accuracy	Recall	Precision	*F*_1_-score
**RoBERTa^a^-large with fine-tuning**		0.76			
	Positive		0.87	0.76	0.82
	Neutral		0.37	0.85	0.52
	Negative		0.94	0.72	0.82
	Average		0.73	0.78	0.72
**GPT-3.5 Turbo without fine-tuning**		0.75			
	Positive		0.83	0.80	0.81
	Neutral		0.48	0.73	0.58
	Negative		0.90	0.73	0.81
	Average		0.74	0.75	0.73
**GPT-3.5 Turbo with fine-tuning**		0.80			
	Positive		0.89	0.81	0.85
	Neutral		0.57	0.74	0.64
	Negative		0.91	0.83	0.86
	Average		0.79	0.79	0.79

^a^RoBERTa: Robustly Optimized BERT Pretraining Approach.

**Table 4:** Sample of tweets classified by GPT-3.5 Turbo with fine-tuning.

Tweets	Score	Sentiment
“I get you. I feel like masks block my turkey gobbler neck, so, sometimes I don't mind wearing them. That, and they keep my face warm in the winter when I go out. 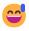 .	–1	Positive
“teaches you how to be an effective, empathetic leader in 0 lessons that you can find at Shot at our Level-0 complex of stages in Sunset Park! 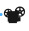 . Tô learn more about our stages, please visit 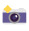 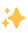 .”	0	Neutral
“I have both vaccines and just got the booster (which takes a while to kick in). Unfortunately, there are a lot of people who go out unmasked when they’re sick and are spreading it like crazy,”	1	Negative

### Result of Sentiment Estimation

#### Overview

To answer RQ3 and RQ4, the sentiment was extracted from posts made between November 28, 2021, and December 31, 2022, in New York City, Los Angeles, and Chicago. Similarly, the number of new cases in New York City, Los Angeles, and Chicago between November 28, 2021, and December 31, 2022, was taken from the New York Times COVID-19 data [39]. The research about the cyclicity of COVID-19 cases reported that in the United States in 2020, the number of new cases was lower on weekends than on weekdays [40]. Therefore, the sentiment was expressed as the weekly arithmetic mean of the sentiment score in each period, which was classified as –1 for positive, 0 for neutral, and 1 for negative. In addition, a 4-week moving average was applied to the sentiment data to smooth out random fluctuations or noise and to account for sentiment trends preceding the number of infected cases [16]. This value is defined as the sentiment index. At the same time, new cases were aggregated weekly to offset the weekend’s effects. The cases were described as a logarithm of 10 to normalize extreme increases due to specific mutant strains. Moreover, these time-series data were plotted on the graph to understand the patterns and trends and to confirm the relevance of each city’s timeline. To quantify the degree and confirm the direction of the linear relationship between the sentiment index and new cases, the correlation coefficient was used to extract the main topics and themes that characterize each period when the number of cases increased or decreased.

#### New York City

**Figure 2:**
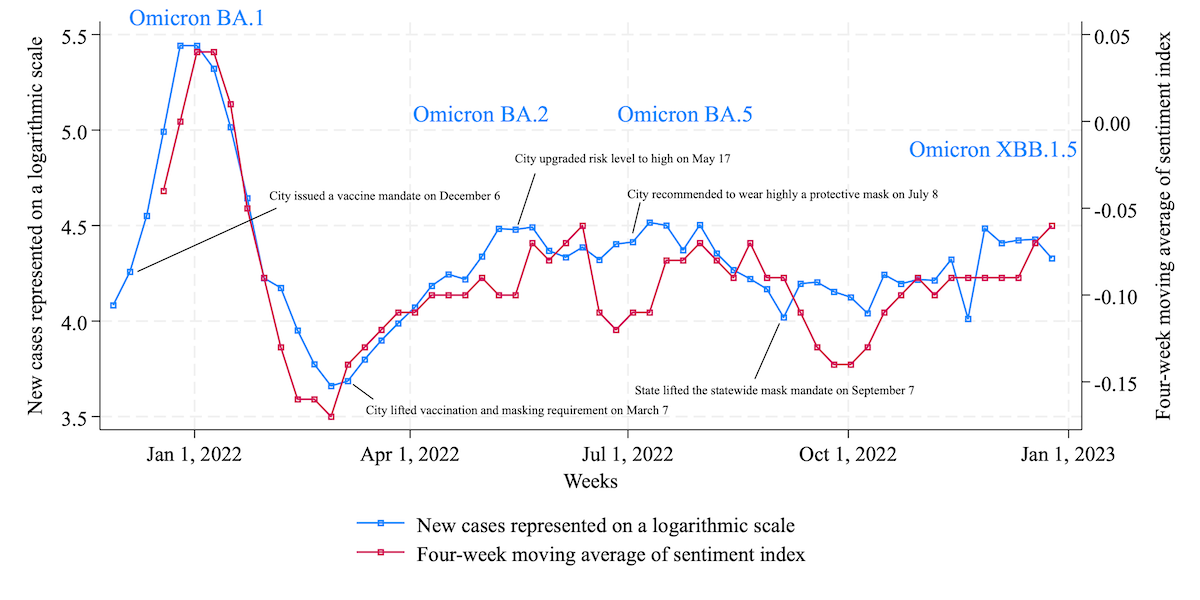
New COVID-19 cases and sentiment index extracted by GPT-3.5 Turbo with fine-tuning in New York City. The closer the sentiment index is to 1, the higher the degree of negativity, while the closer it is to −1, the higher the degree of positivity.

The left axis of Figure 2 shows the number of cases represented by a logarithmic scale in New York County, New York, and the right axis shows the average sentiment over 4 weeks extracted by GPT-3.5 Turbo fine-tuned with training data. In December 2021, citizens in New York were exposed to a surge in Omicron variant [41] cases and subsequently experienced cyclical waves of infections from subvariants such as Omicron BA.2 [42] and Omicron XBB.1.5 [43], which became predominant, particularly in the Northeast, by January 2023. Unlike in 2020, when COVID-19 had a high death rate, measures such as stay-at-home orders, bans on large gatherings, and travel restrictions were not enforced by the state government during this period, although mandates such as vaccinations [44] for specific workers and indoor mask wearing [45] in clinics were implemented or recommended.

Here, we performed a statistical test on the sentiment waveform. As shown in Table 5, the correlation coefficient between sentiment and cases is 0.89, indicating a strong positive relationship. This suggests that in New York City, the sentiment of posts related to pandemic restrictions was consistently associated with COVID-19 infection status throughout the period. The correlation between the sentiment of posts related to restrictions on gatherings and cases was relatively weaker at 0.40 than for other types. There is a possibility that certain anxiety about commuting to work, going to school, and traveling may have persisted due to the spread of COVID-19, but it can be assumed that citizens exercised less caution when it came to leisure activities such as going to the movies, going on dates, and shopping.

Table 6 shows the feature words extracted using term frequency–inverse document frequency (TF-IDF) for each period according to the infection status. During December 2021, when infections caused by the Omicron strain spiked, themes related to polymerase chain reaction tests and the Omicron strain were dominant, whereas from January 2022 onward, concerns about COVID-19 ceased to be the main topic. Although Table 5 confirms a strong relationship between sentiment and infection status, the number of posts using keywords related to COVID-19 decreased from the beginning of 2022.

**Table 5:** The correlation coefficient between new COVID-19 cases and sentiment index extracted by GPT-3.5 Turbo with fine-tuning from December 2021 to December 2022.

			*df*^a^ (cases and index pairs)		Average log cases^b^		Average index^c^		*r*^d^ (95% CI^e^)		*P* value
**New York City**											
	Total	52		4.3		–0.1		0.89 (0.81 to 0.93)		<.001	
	Stay-at-home order	52		4.3		0.2		0.69 (0.41 to 0.76)		<.001	
	Restrictions on gatherings	52		4.3		–0.1		0.40 (0.15 to 0.61)		<.001	
	Travel restrictions	52		4.3		–0.2		0.73 (0.58 to 0.84)		<.001	
**Los Angeles**											
	Total	52		4.3		–0.1		0.39 (0.14 to 0.60)		<.001	
	Stay-at-home order	52		4.3		0.2		0.41 (0.17 to 0.61)		<.001	
	Restrictions on gatherings	52		4.3		−0.1		0.13 (−0.15 to 0.38)		<.001	
	Travel restrictions	52		4.3		–0.3		0.52 (0.29 to 0.69)		<.001	
**Chicago**											
	Total	52		4.0		–0.1		0.65 (0.47 to 0.78)		<.001	
	Stay-at-home order	52		4.0		0.1		0.63 (0.43 to 0.77)		<.001	
	Restrictions on gatherings	52		4.0		–0.1		0.42 (0.18 to 0.62)		<.001	
	Travel restrictions	52		4.0		–0.3		0.48 (0.24 to 0.66)		<.001	

^a^*df* calculated as n – 2, where n is the number of paired observations.

^b^Avg log cases: the average of the logarithmically transformed number of new COVID-19 cases that were summarized on a weekly basis.

^c^Avg index: the average sentiment index derived from GPT-3.5 Turbo with fine-tuning that was summarized on a weekly basis.

^d^The correlation coefficient between average log cases and average index.

^e^95% CI for the correlation coefficient.

**Table 6:** Feature words extracted by term frequency–inverse document frequency in New York City, Los Angeles, and Chicago.

Period			Trend^a^		Feature words
**New York City**					
	December 2021 to January 2022	Rise^b^		“nye”, “santa”, “*tests*”, “*cases”*, “*pcr”*, “xmas”, “eve”, “playmates”, “*omicron”*, “citymd”	
	January 2022 to March 2022	Fall^c^		“adon”, “magazine”, “partnership”, “snow”, “playmates”, “jorge”, “cric”, “holly”, “ostine”, “har”	
	March 2022	Rise		“adon”, “magazine”, “partnership”, “mfa”, “thesis”, “easter”, “ukraine”, “ecea”, “abortion”, “ladies”	
	May 2022 to June 2022	Fall		“gelato”, “abortion”, “juneteenth”, “britney”, “Imran”, “kele”, “sonny”, “ceremony”, “conference”	
	June 2022 to August 2022	Rise		“taiwan”, “pelosi”, “gelato”, “abortion”, “gyamfua”, “teresa”, “dj”, “speaker”, “supplies”, “nancy”	
	August 2022 to October 2022	Fall		“supplies”, “hurricane”, “marcos”, “dj”, “princess”, “backpacks”, “zuccotti”, “sp”, “sept”, “depot”	
	October 2022 to December 2022	Rise		“thanksgiving”, “exile”, “election”, “pt”, “santa”, “comics”, “thankful”, “profile”, “mets”, “feet”	
**Los Angeles**					
	December 2021 to January 2022	Rise		“shooky”, “*omicron”*, “mang”, “xmas”, “rain”, “nye”, “*tested”*, “eve”, “*vaccinated”*, “pickup”	
	January 2022 to February 2022	Fall		“meye”, “spotify”, “launch”, “vibration”, “return”, “colors”, “reach”, “train”, “katelyn”	
	February 2022 to March 2022	Rise		“drumz”, “ikeboy”, “corey”, “chorus”, “braves”, “platforms”, “speed”, “putin”, “pickup”	
	March 2022 to April 2022	Fall		“anaheim”, “katani”, “axie”, “mace”, “wednesdays“, “mombasa”, “syokimau”, “rd”, “gig”	
	April 2022 to June 2022	Rise		“gun”, “train”, “demand”, “mass”, “lots”, “easter”, “stand”, “anaheim“, “responders”, “dodger”	
	June 2022 to July 2022	Fall		“kbla”, “abortion”, “kele”, “tumi”, “calhope”, “inquiries”, “usc”, “alex”, “folks”, “lots”	
	July 2022 to November 2022	Rise		“halloween”, “return”, “lots”, “train”, “sinners”, “folks”, “stand”, “sharing”, “bday”, “sept”	
	November 2022 to December 2022	Rise		“thanksgiving”, “xmas”, “return”, “folks”, “rain”, “lots”, “picture”, “code”, “oregon“, “stub”	
**Chicago**					
	December 2021 to January 2022	Rise		“christmas”, “*testing”*, “*vaccinated”*, “*vaxxed”*, “winter”, “merry”, “*tested”*, “teachers”, “*tests”*, *“masks”*	
	January 2022 to February 2022	Fall		“snow”, “polio”, “valentines”, “n”, “mae”, “quite”, “*vaccine”*, “lens”, “strap”, “rethink”	
	February 2022 to March 2022	Rise		“ukraine“, “creature”, “ukrainian”, “women”, “session”, “uppf”, “group”, “required”, “*vaccine”*, “*masks”*	
	March 2022 to May 2022	Fall		“orvieto”, “pauline“, “uppf”, “pagbalik”, “ni”, “fralaine”, “writers”, “wines”, “nevada”, “celebrate”	
	May 2022 to May 2022	Rise		“nra”, “kardashian“, “kourtney”, “send”, “lee”, “government”, “tragedy”, “travis”, “robb”, “families”	
	May 2022 to June 2022	Fall		“national”, “women”, “final”, “logical”, “bush”, “solution”, “event”, “tourists”, “crops”, “alexander”	
	June 2022 to July 2022	Rise		“women”, “abortion”, “highland”, “harris”, “tamil”, “celebrate”, “tornado”, “session”, “send”	
	July 2022 to August 2022	Fall		“bash”, “supplies”, “finkl”, “saudi“, “group”, “biden”, “bag”, “faculty”, “lee”, “jaz”	
	August 2022 to October 2022	Rise		“bark”, “tickets”, “birthday”, “dance”, “dress”, “event”, “n”, “hc”, “downtown”, “celebrate”	
	October 2022 to November 2022	Fall		“halloween”, “dailey”, “juniors”, “sign”, “visiting”, “encouraged”, “hermanos“, “fascism”, “attend”	
	November 2022 to December 2022	Rise		“christmas”, “thanksgiving”, “santa”, “winter”, “merry”, “snow”, “tickets”, “gift”, “storm”	

^a^Trend denotes the current infection status of COVID-19. “Rise” signifies the period of infection expansion, and “Fall” indicates the decline or conclusion of the infection period.

^b^Rise signifies the period of infection expansion.

^c^Fall indicates the decline or conclusion of the infection period. Italicized texts in the Feature words column represent words relevant to COVID-19.

#### Los Angeles

**Figure 3:**
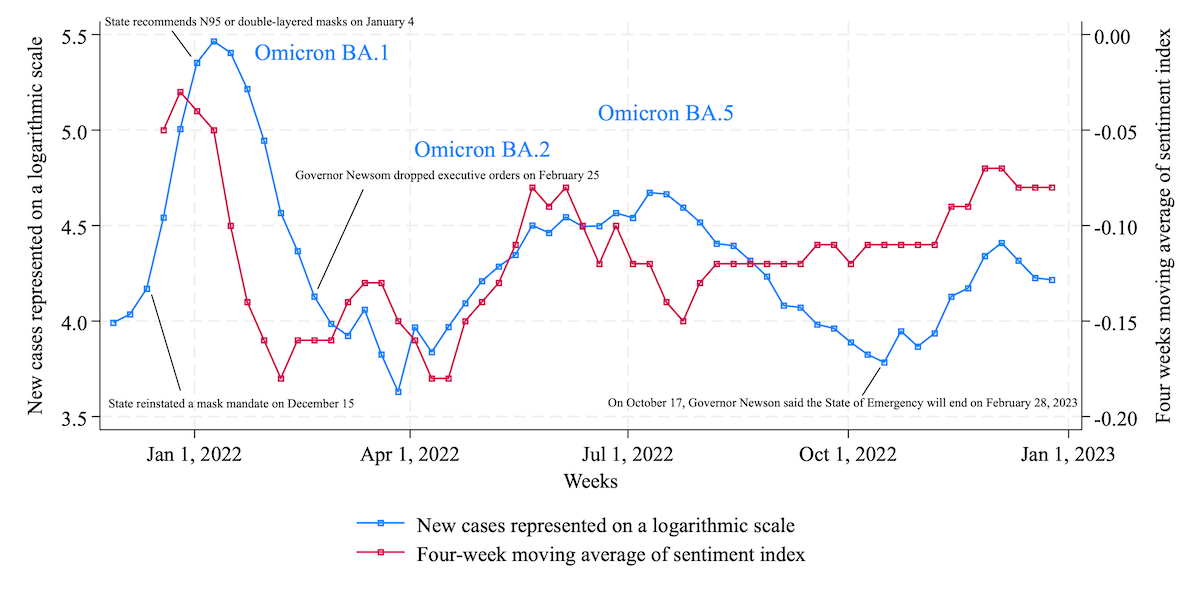
New COVID-19 cases and sentiment index extracted by GPT-3.5 Turbo with fine-tuning in Los Angeles.

The left axis of Figure 3 shows the number of cases represented by a logarithmic scale in Los Angeles County, California, and the right axis shows the average sentiment over 4 weeks extracted by GPT-3.5 Turbo fine-tuned with training data. Similar to New York City, Los Angeles experienced an increase in Omicron infections in December 2021 [46], followed by subvariant BA.2 infections in spring 2022 [47] and subvariant BA.5 infections in summer 2022 [48]. Unlike in New York City, cases caused by the Omicron subvariant XBB.1.5 were limited in Los Angeles in December 2022 [49]. During this period, as in New York City, the state government did not order any restrictions on public movements, mainly requiring masks in public indoor settings [50] and recommending vaccinations for health care workers [51].

Here, we examine the correlation between sentiment and cases from Table 5. For the total sentiment index and cases pairs, *r* is 0.39, which is lower than that in New York City (*r*=0.89). However, by setting a 2-week lag in cases in the correlation coefficient, it was confirmed that *r* was 0.61 (*P*<.001). From this, it can be confirmed that in Los Angeles, sentiment preceded infection status. Similar to New York City, there was a relatively high correlation (0.52) between posts related to travel restriction and infection status and a low correlation (0.13) between posts related to restrictions on gatherings and infection status. In addition, as shown in Figure 3, it is notable that although the number of cases decreased since fall 2022, sentiment did not move positively as it did in spring 2022.

Table 6 shows the feature words extracted for each period according to infection status. In late December 2021 when infections caused by the Omicron strain rose, words related to Omicron and vaccination became common. However, from January 2, 2022, words related to COVID-19 dropped from the top of the TF-IDF scores, following the same trend observed in New York City. This means that although subvariants such as BA.2 and BA.5 appeared after January 2022, public interest in COVID-19 had waned.

#### Chicago

**Figure 4:**
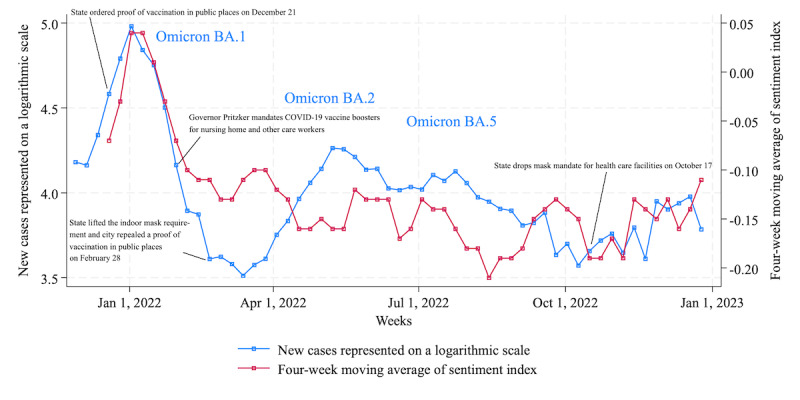
New COVID-19 cases and sentiment index extracted by GPT-3.5 Turbo with fine-tuning in Chicago.

The left axis of Figure 4 shows the number of cases represented by a logarithmic scale in Cook County, Illinois, and the right axis shows the average sentiment over 4 weeks extracted by GPT-3.5 Turbo fine-tuned with training data. In Chicago as well, after the surge of Omicron infection at the end of 2021 [52], subvariant BA.2 became dominant in spring [53], and BA.5 replaced it in summer [54]. In Illinois, from December 2021 to 2022, vaccination and mask mandates were intermittently issued for specific situations and workers, but as in New York City and Los Angeles, restrictions on movements were also imposed.

As shown in Table 5, the correlation coefficient between cases and sentiment in Chicago was 0.65 throughout the period, which is lower than that in New York City (0.89) and higher than that in Los Angeles (0.39). Regarding the correlation between cases and sentiment, unlike New York City and Los Angeles, there was a relatively higher correlation (0.63) between sentiment related to stay-at-home orders and cases. In Chicago, concerns about going to school or work may have been higher than concerns about social activities and travel.

Table 6 shows the feature words extracted for each period according to infection status. Unlike New York City and Los Angeles, we can see that keywords related to COVID-19 were common until March 2022. In Chicago, the indoor mask mandate was lifted [55], and proof of vaccination in public places was repealed on February 28 [56], but Chicagoans may have had a more sustained interest in wearing masks and getting vaccinated compared with residents in the other 2 cities.

## Discussion

### Principal Findings

Considering the findings presented in the Results section, here we address RQ3 and RQ4. First, we undertake RQ3: “To what extent will the cyclical sentiment of citizens toward restricted activities in major US cities during the ‘new normal’ period weaken over time following its peak in December 2021?.” Our results indicate a decrease in negative sentiment starting in April 2022, as evidenced by the peak of negative sentiment being below –0.05 in New York City (Figure 2) and below –0.1 in Chicago (Figure 4), despite the emergence of additional variants such as Omicron BA.2 and BA.5. In contrast to New York City and Chicago, sentiment in Los Angeles remained consistently within 75% of the initial peak observed during the week of December 26, 2021, maintaining this level from August 2022 onward (Figure 3). This phenomenon can be attributed to the relatively modest peak value (–0.03) for Los Angeles compared with the peak values of 0.03 in New York City and 0.04 in Chicago during the weeks of January 2 and January 9, 2022. Overall, we confirmed that the tone of the sentiment toward restricted social activities changed from negative to moderate throughout 2022, a trend observed consistently across these 3 US cities. According to the investigation about prolonged pandemic symptoms, significantly lower levels of limiting one’s activities were reported among adults aged 18 to 39 years in the United States in June 2022 compared with other age groups [57]. This age group overlapped with the Twitter user group [58], partially supporting the sentiment estimation results.

Second, we address RQ4: “In comparing citizens’ sentiments across major metropolitan areas in the United States, namely, New York City, Los Angeles, and Chicago, between December 2021 and December 2022, what differences or similarities in sentiment were there?” In terms of sentiment, the correlation coefficient between New York City and Los Angeles was 0.64, between Los Angeles and Chicago was 0.32, and between Chicago and New York City was 0.60, indicating an overall positive correlation. In feature words extracted by TF-IDF, during the peak of the initial Omicron wave in New York City and Los Angeles, feature words associated with COVID-19 were common; however, they were notably absent in subsequent periods. Meanwhile, in Chicago, feature words related to COVID-19 remained prominent in Twitter’s message space until March 2022, indicating sustained interest in COVID-19. From then on, COVID-19–related keywords in each city were replaced by words from political contexts such as “Ukraine” and “abortion,” weather-related words such as “hurricane” and “rain,” and other words with unknown contexts. The abovementioned results are consistent with the trend of weakening sentiment throughout 2022.

Third, we consider the methodology. A novel aspect of our study is the accuracy of the sentiment estimation model, which was developed using Twitter posts extracted under the same conditions as the observed message group focused on COVID-19 awareness among people in 2021. Previous studies relied on data from before 2020, when COVID-19 emerged in the sentiment classification model. In such cases, a domain shift problem is likely to occur between the registered lexicon or training data and the observed data, which would affect the accuracy of the results. This problem remains an issue to be addressed, from conventional neural networks to LLMs, and technical solutions are being discussed [59,60]. We overcame these issues by training the model explicitly on data from the COVID-19 period.

### Comparison With Prior Work

We compare our results with those of previous studies. The novelty of this study is that it showed a positive correlation over time between sentiment and cases during the “new normal” period. In a similar study, a survey in the Greater London area delved into the recovery of sentiment at the city level during the COVID-19 pandemic and found a gradual recovery in sentiment over time after reopening, similar to the findings of this study [61]. However, this study merely confined the visualization of sentiment estimation results to their association with reopening events and did not reveal a correlation with any variables related to COVID-19. In the prior study, sentiment transitioned positively with a delay after reopening events, whereas in our study, sentiment shifted positively concurrently with the issuance of reopening orders. The difference in these results is thought to stem from whether the observed messages associated with keywords related mainly to COVID-19 or to social activities restricted by the pandemic.

Next, we refer to the previous study conducted by the author [16], which explored sentiment in New York City, Los Angeles, and Chicago from December 2019 to December 2021, using data collected under the same conditions as in this study. The sentiment waveforms correlated with infection status show the same trend, but the crucial difference is the tendency of feature words related to COVID-19 to appear. Both papers show that feature words related to COVID-19 had high TF-IDF values in New York City and Los Angeles from March 2020 to January 2022 and in Chicago from March 2020 to March 2022. In contrast, after the aforementioned period, feature words associated with COVID-19 did not surface among the top rankings in these 3 cities. In the context of COVID-19, it is plausible that Twitter’s message space reverted to a state akin to that of February 2020 after the spring of 2022.

### Limitations

It is important to acknowledge certain limitations of our study. First, Twitter users in the United States have tended to skew toward the 18- to 39-year age group, which constitutes 62% of the user base [58]. Moreover, there was a notable gender bias, with males accounting for 63% of the total users [62]. It is essential to recognize that these demographics may not perfectly align with the overall population distribution in the United States. Consequently, our study’s observed data may exhibit a slight bias toward younger male demographics. Second, in this study, we estimated citizen sentiment by focusing on activities that were restricted during the COVID-19 pandemic, but these may not necessarily be messages posted in the context of the pandemic. As the goal of this study was to capture the societal atmosphere during the “new normal” period, we did not intend to limit our observations to the context of the COVID-19 pandemic. However, it is important to keep this context in mind as a premise.

### Conclusions

This study estimated citizens’ sentiment in major cities in the United States, spanning the period from the exponential surge in COVID-19 infections to the gradual abatement of the pandemic as it approached its conclusion. This research makes 3 primary contributions. First, it enhances the performance of the sentiment classifier by fine-tuning the LLM using data under the same conditions as the observed target. The efficacy of this improvement was validated through a comparison with previous models using *F*_1_-score metrics. Second, our findings revealed a positive correlation between the estimated sentiment of citizens and actual cases of COVID-19 in New York City, Los Angeles, and Chicago. Although variations exist among these cities, a consistent trend emerged showing a gradual decline in sentiment, which corresponded to a reduction in the number of infections. Third, across these cities in 2022, references to COVID-19 gradually disappeared in the context of restricted social activities as the pandemic neared its end. This phenomenon is evidenced by the disappearance of COVID-19–related words in feature words after the spring of 2022.

The observational data design and sentiment classifier creation method used in this study have potential applications beyond the context of the COVID-19 pandemic. They can be adapted to address various other crises that humanity encounters intermittently, including infectious diseases, natural disasters, terrorism, and warfare. In the future, this field of study is expected to evolve and be applied more broadly to society, supported by stronger collaboration between social science and computer science.

## Appendix

Multimedia Appendix 1 Keywords related to citizens’ activities that were constrained by lockdowns and similar measures implemented in response to the COVID-19 pandemic.

## Abbreviations

API: Application Programming Interface
BERT: Bidirectional Encoder Representations from Transformers
LLM: large language model
MTurk: Mechanical Turk
NLP: natural language processing
RoBERTa: Robustly Optimized Bidirectional Encoder Representations from Transformers Pretraining Approach
RQ: research question
TF-IDF: term frequency–inverse document frequency
