# Supplementary material for: Understanding Citizens’ Response to Social Activities on Twitter in US Metropolises During the COVID-19 Recovery Phase Using a Fine-Tuned Large Language Model: Application of AI
Source: J Med Internet Res. 2025 Feb 11;27:e63824. doi: 10.2196/63824 (PMC11862765; doi:10.2196/63824)
Supplement: Multimedia Appendix 1 [file jmir_v27i1e63824_app1.pdf]

Table S1. Keywords related to citizens' activities that were constrained by lockdowns and similar measures implemented in response to the COVID-19 pandemic

Restrictions		Keywords	
Stay-at-home order		go(-ing) out	to college
		go(-ing) to work	attend the class
		to my office	by train
		to school	by bus
Restrictions on gatherings	Public event	to the movies	gathering
		to the theater	to a club
		see a play	to the amusement[a]
		to a (the) concert	go bowling
		to the gig	to casinos
		to the museum	to the racetrack
		to the planetarium	to the bingo[b]
		to the auditorium	to an (the) arcade
		see the game	to church
		to the stadium	
	Private event	wedding	to a bar
		on a date	get-together
		to a (the) party	drinking party
		have a party	to a restaurant
		to bbq	eat(-ing) out
		have a bbq	to a café
		for a drink	meetup
	Non-essential retail	go(-ing) shopping	go swimming
		to the mall	to a (the) spa
		to the salon	to yoga
		get a haircut	to massage
		to the gym	
Travel restrictions		travel(-ing)	by plane
		on a trip	visit
		fly to	to my hometown

[a]Assuming "to the amusement parks".

[b]Assuming "to the bingo halls".
